# SCoVMod – a spatially explicit mobility and deprivation adjusted model of first wave COVID-19 transmission dynamics

**DOI:** 10.12688/wellcomeopenres.17716.1

**Published:** 2022-05-26

**Authors:** Christopher J. Banks, Ewan Colman, Thomas Doherty, Oliver Tearne, Mark Arnold, Katherine E. Atkins, Daniel Balaz, Gaël Beaunée, Paul R. Bessell, Jessica Enright, Adam Kleczkowski, Gianluigi Rossi, Anne-Sophie Ruget, Rowland R. Kao

**Affiliations:** 1 Roslin Institute, University of Edinburgh, Edinburgh, EH25 9RG, UK; 2 Mathematics & Statistics, University of Strathclyde, Glasgow, G1 1XH, UK; 3 The Animal and Plant Health Agency, Weybridge, Surrey, KT15 3NB, UK; 4 Faculty of Epidemiology and Population Health, on School of Hygiene and Tropical Medicine, WC1E 7HT, UK; 5 Usher Institute, University of Edinburgh, Edinburgh, EH8 9AG, UK; 6 INRAE, Nantes, 44300, France; 7 School of Computing Science, University of Glasgow, Glasgow, G12 8RZ, UK; 8 Royal (Dick) School of Veterinary Studies, University of Edinburgh, Edinburgh, EH25 9RG, UK

**Keywords:** COVID-19, epidemic, spatio-temporal, model, deprivation, mobility, health

## Abstract

**Background**: Mobility restrictions prevent the spread of infections to disease-free areas, and early in the coronavirus disease 2019 (COVID-19) pandemic, most countries imposed severe restrictions on mobility as soon as it was clear that containment of local outbreaks was insufficient to control spread. These restrictions have adverse impacts on the economy and other aspects of human health, and it is important to quantify their impact for evaluating their future value.

**Methods**: Here we develop Scotland Coronavirus transmission Model (SCoVMod), a model for COVID-19 in Scotland, which presents unusual challenges because of its diverse geography and population conditions. Our fitted model captures spatio-temporal patterns of mortality in the first phase of the epidemic to a fine geographical scale.

**Results**: We find that lockdown restrictions reduced transmission rates down to an estimated 12\% of its pre-lockdown rate. We show that, while the timing of COVID-19 restrictions influences the role of the transmission rate on the number of COVID-related deaths, early reduction in long distance movements does not. However, poor health associated with deprivation has a considerable association with mortality; the Council Area (CA) with the greatest health-related deprivation was found to have a mortality rate 2.45 times greater than the CA with the lowest health-related deprivation considering all deaths occurring outside of carehomes.

**Conclusions**: We find that in even an early epidemic with poor case ascertainment, a useful spatially explicit model can be fit with meaningful parameters based on the spatio-temporal distribution of death counts. Our simple approach is useful to strategically examine trade-offs between travel related restrictions and physical distancing, and the effect of deprivation-related factors on outcomes.

## Introduction

The coronavirus disease 2019 (COVID-19) pandemic resulted in the introduction of severe non-pharmaceutical interventions (NPIs) to control its spread in most countries around the world. COVID-19 was introduced into Scotland no later than February 2020. Following a series of lesser restrictions and recommendations, on 23rd March a *lockdown* with widespread NPIs was put in place across the UK, which resulted in cessation of all non-essential activities, many businesses closed and most individuals except *key workers* were restricted to only short distance trips from their homes, initially with a maximum frequency of only once per day. These measures reduced the transmission of COVID-19 substantially, so that by the first week of April, the average infected individual in Scotland was estimated to be infecting fewer than one other individual each (i.e. causing the reproduction number R to fall below one).

While we therefore know that the combination of measures was effective, no study as yet has directly estimated the impact of different aspects of these control policies. One factor that makes this difficult for the evaluation of any such national level policy is the impact of variation in space of the disease transmission itself. For COVID-19, one critical issue is the known substantial variation in COVID-19 infection and mortality risk associated with deprivation ^1^. The impact of deprivation is likely due to a combination of factors influencing both exposure (e.g. more crowded housing and working conditions) and mortality once exposed (e.g. due to already poorer health) and this should be accounted for in any assessment of the impact of restrictions.

Here, we develop an explicitly spatial agent-based simulation model that accounts for recorded movements-to-work (i.e. “commuter” patterns), modulated by recorded time-varying mobility statistics, and geographically explicit population age structures. Whilst this does not capture all human movement, it is expected to capture a large proportion of the long-range mobility especially affected by lockdown. We also modify the modelled epidemic using deprivation metrics. In these early epidemic stages case data were unevenly recorded, therefore we use this model to estimate transmission characteristics by fitting it to the observed number of COVID-19 related deaths using Approximate Bayesian inference. Our aim is to estimate the impact of travel restrictions and transmission reduction on the spread of COVID-19, and to assess the impact of measures of deprivation in order to estimate its impact on COVID-19 related mortality.

The impact of NPIs is of considerable interest and has been the subject of several analyses, however previous results have focused on statistical comparisons of interventions across multiple countries ^2– 4^. Such approaches are extremely useful for identifying the impact of events, but do not directly allow for disentangling the impact of simultaneous events. Long distance movement and local restrictions are typically applied contemporaneously, and our approach that includes processes at the two scales (local and regional) allows us to estimate the impact of each.

## Methods

### Data sources

We use a combination of publicly available Scottish census data from National Records for Scotland (NRS) ^1^ and data on COVID-19 held by Public Health Scotland (PHS) and made available via the PHS Electronic Data Research and Innovation Service (eDRIS) ^2^. We used data zone (DZ) level resolution where DZs are population census units of approximately 500 to 1,000 residents. The data for assignment of individuals to work locations is drawn from the NRS Census Flows data ^3^, Table WU01UK, which provides origin/destination workplace data for the population from the 2011 census. We adjust these with respect to the 2018 population estimates.

Age demographics and movement to work patterns are available at the level of Census Output Areas (OA), each of which contains approximately 20 households or 50 people ^4^.

Census data on the Scottish Index of Multiple Deprivation (SIMD) ^5^ considers multiple relative deprivation measures and combines them into a single value. Deprivation data are publicly available at the DZ level.

We also used publicly available data from Google to estimate mobility levels over time, with respect to commuting patterns ^6^.

### Model overview

The model we present here breaks down into a number of distinct parts, each of which we describe over the following sections. The code is available from GitHub and is archived with Zenodo. First, to account for the variation in mortality risk due to deprivation factors we develop a statistical model that provides a mortality parameter adjustment to the main model. The core of the simulation model then breaks down into the following parts:

- Local transmission—a homogeneous mixing compartmental model for each unit area of the country;
- National transmission—a network-based simulation of the movement of individuals between unit areas;
- Lockdown simulation—the reduction of both local and national transmission to simulate non-pharmaceutical interventions;
- Parameter inference—a Bayesian estimation of the parameters for local transmission.

**Figure 1.**
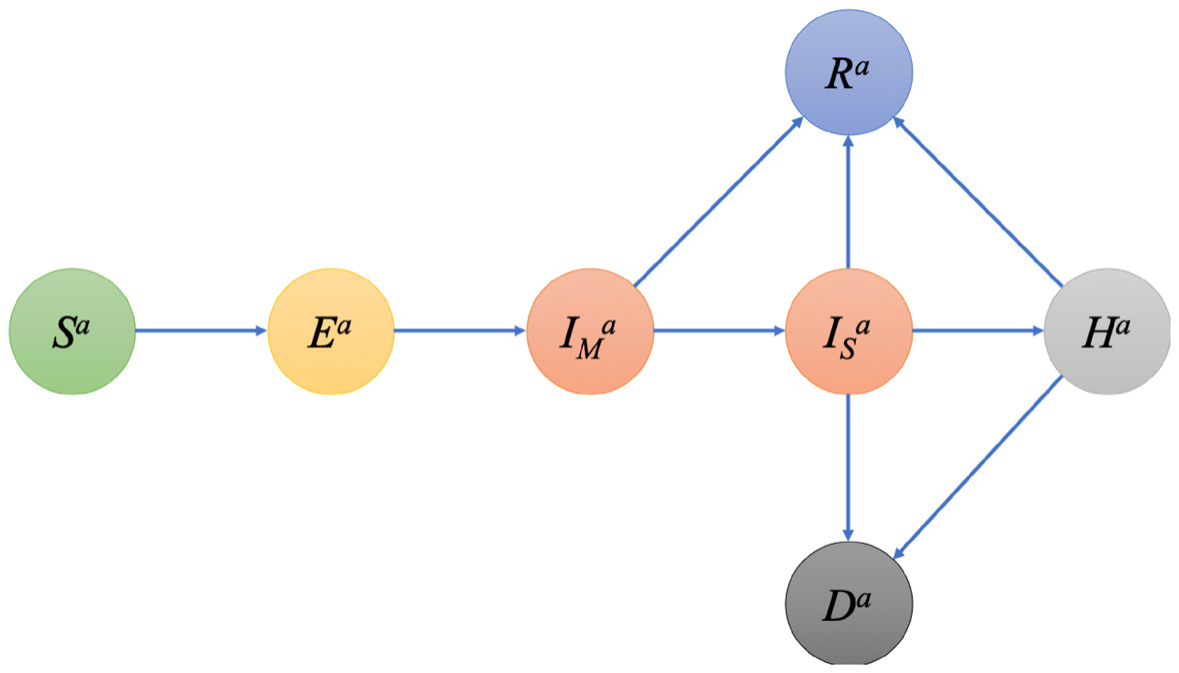
Schematic of infection stages in SCoVMod. Individuals pass through stages post infection as described by arrows. Not all stages are obligatory for all infected individuals (e.g. some individuals recover without going to hospital). SCoVMod, Scotland Coronavirus transmission Model.

The simulation framework (Scotland Coronavirus transmission Model, or SCoVMod) considers key aspects of COVID-19 epidemiology including phases for latent infection, infectious and mildly infected (showing few or no clinical signs) and severely infected (with substantial clinical signs) individuals, hospitalised, recovered and died, similar to other investigations ^5, 6^. These epidemiological processes are captured as individual disease states ( Figure 1). Individuals are also stratified into three age groups: young (0–15), adult (16–64) and elderly (65+). Within-OA transmission is assumed to be homogeneously mixed, while between-OA transmission is determined by the empirical age-specific patterns of home and work contact (creating day/night patterns of contact). We do not consider overnight shifts in location or introductions from outside Scotland beyond the impact on the initial seeding. Death rates for all age classes are assumed to be the same, but with differing recovery rates, resulting in age-dependent differences in the infection-related mortality, as is consistent with the data at the time (see Table 1). Deprivation is also known to influence COVID-19 mortality ^7^; we therefore adjust mortality in the model with the average health index in the local council area (see below).

**Table 1.** Epidemiological parameters in SCoVMod, with priors and fixed values as appropriate. Where age is not indicated, parameters are assumed to be age independent. All times are measured in days. SCoVMod, Scotland Coronavirus transmission Model; eDRIS, Electronic Data Research and Innovation Service.

Parameter	Transition	Symbol	Age	Value	Prior	References
Latency period	*E* → *I ^M^*	1 */γ*	All	fitted	U(1.67,28)	5, 9– 12
Days from mild infectiousness to recovery	*I ^M^* → *R*	1 */ρ _M_*	All	fitted	U(0.67,28)	6, 12
Symptom onset time after infectiousness	*I ^M^* → *I ^S^*	1 */γ _M_*	All	fitted	U(2,28)	5, 9– 16
Transmission rate for severe infectors (baseline, daytime)	*S* → *E*	*β _d_*	All	fitted	U(0,2.8)	
Transmission rate for severe infectors (baseline, nightime)	*S* → *E*	*β _n_*	All	fitted	U(0,2.6)	
Transmission rate multiplier for mild infectors	*S* → *E*	*y*	All	fitted	U(0,2.6)	
Severe symptom onset to hospitalization	*I ^S^* → *H*	1 */η*	All	4		15– 22
Severe symptom onset to recovery for non-hospitalised	*I ^S^* → *R*	1 */ρ _S_*	Young Adults Elderly	19 20.7 21.6		15
Days hospitalisation to death	*H* → *D*	1 */µ _H_*	Young Adults Elderly	6.97 6.62		eDRIS data eDRIS data eDRIS data
Proportion of hospitalised who recover	*H* → *R*	*ρ _H_ /*( *ρ _H_* + *µ _H_* )	Young Adults Elderly	1 0.96 0.84		eDRIS data eDRIS data eDRIS data
Symptoms onset to death	*I ^S^* → *D*	1 */µ _S_*	Adults	16		16, 18, 21– 24
Mortality rate multiplier (relative to average health index)		*µ _mod_*	All	fitted	U(0,0.08)	
Number of seed infections		*N _s_*	N/A	fitted	U(0, 2000)	

We make a number of simplifying assumptions regarding transmission pathways. First, infections in care homes are not modelled at this stage of the epidemic, as they are assumed to result in few additional infections outside of these locations. Second, hospital-acquired infections are considered ‘dead ends’, following evidence that they do not result in substantial community outbreaks ^8^. Third, population mobility patterns are determined by the patterns of movements to work, recorded in Scottish Census data. We assume that only adults contribute to commuter movement, in the daytime; the remaining proportion of adults and all young and elderly individuals are assumed to move primarily within their local OAs, which also account for non-work activities. Finally, commuting is restricted to healthy and exposed or mildly symptomatic individuals; severely infected and hospitalised individuals do not commute. The day/night patterns also result in two transmission rates.

### Health index and mortality

Using the SIMD we constructed two initial statistical models, with a view to guiding the parameters of the core simulation model. These consisted of:

1. A population level mortality model
2. An excess deaths model

The models were generalised linear models with binomial error structures at the local Council Area (CA) level (N=32). For the population level model the outcome variable was c(Covid deaths, population – Covid deaths). For the excess mortality model c(Covid deaths, all deaths – Covid deaths). To correct for overdispersion we fitted the model with an individual level random effect for each data point using the glmer function in the lme4 package for R (R package: lme4, RRID:SCR_015654), in multivariable models variance inflation factors were checked using the car package (R package: car, RRID:SCR_022137) and overdispersion using the DHARMa package (R package: DHARMa, RRID:SCR_022136). We also checked the results against a model fitted using the quasi-binomial family. In both models we tested the following covariates in univariable analysis: Population (from SIMD 2020), Population density, SIMD 2016 score, score without the access component, income indicator, employment indicator, health indicator, education indicator, housing indicator, access indicator, and crime indicator.

### Local transmission (within-OA)

**Figure 2.**
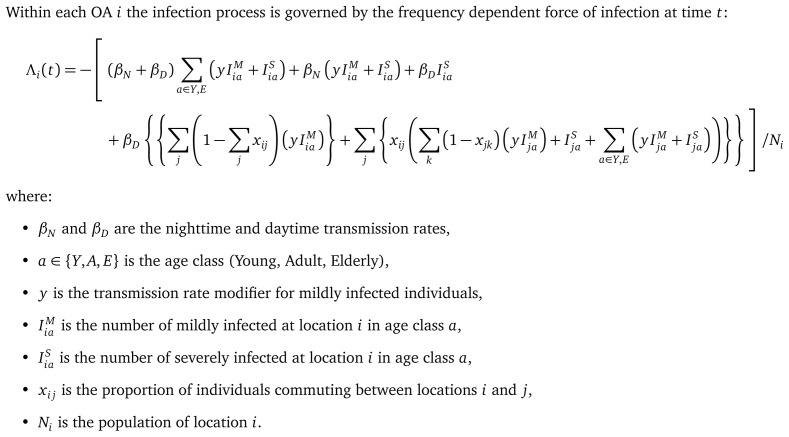
Equation: Force of infection for location *i* at time *t*.

Within each OA ( *i*) the infection process is governed by a compartmental model for which the frequency dependent force of infection Λ _*i*_( *t*) defined in Figure 2. In the compartmental model are infection classes *S* (susceptible), *E* (exposed), *I ^M^* (mildly infected), *I ^S^* (severely infected), *H* (hospitalised). Model equations for individuals residing in one OA *i* and for age class *a* are therefore:


$$\begin{array}{l} \frac{dS_{ia}}{dt}=\text{−}{\text{Λ}}_{i}(t)S_{ia}\\ \frac{dE_{ia}}{dt}={\text{Λ}}_{i}(t)S_{ia}-\gamma E_{ia}\\ \frac{dI_{ia}^{M}}{dt}=\gamma E_{ia}-(\gamma_{M}+\rho_{M})I_{ia}^{M}\\ \frac{dI_{ia}^{S}}{dt}=\gamma_{M}I_{ia}^{M}-(\rho_{Sa}+\mu_{Sia}+\eta )I_{ia}^{S}\\ \frac{dH_{ia}}{dt}=\eta I_{ia}^{S}-(\rho_{Ha}+\mu_{Hia})I_{ia}^{S}\\ \frac{dR_{ia}}{dt}=\rho_{M}I_{ia}^{M}+\rho_{Sa}I_{ia}^{S}+\rho_{Ha}H_{ia} \end{array}$$

The number of deaths is determined by:


$$D_{ia}=N_{ia}-(E_{ia}+I_{ia}^{M}+I_{ia}^{S}+H_{ia}+R_{ia})$$

The rates of infection are detailed in Figure 2 and other state transition rates are given by:

- *γ* for *E* → *I ^M^*
- *γ _M_* for *I ^M^* → *I ^S^*
- *η* for *I ^S^* → *H*
- *ρ _M_* for *I ^M^* → *R*
- *ρ _Sa_* for *I ^S^* → *R* for age class *a*
- *ρ _Ha_* for *H* → *R* for age class *a*
- *μ _Sia_* for *I ^S^* → *D* for age class *a* and location *i*
- *μ _Hia_* for *H* → *D* for age class *a* and location *i*

Mortality rates are adjusted by location, according to Health Index (see results of Health Index and Mortality):


$$\mu_{CA}=\mu_{av}\mu_{mod}\left(1+\left(\frac{k_{CA}-k_{av}}{k_{av}}\right)\right)$$

where *μ _CA_* is the COVID-19 related mortality rate for a given CA, *μ _av_* is the average across CAs, *k _CA_* is the CA mean health index value (from the SIMD), and *μ _mod_* is a fitted parameter given a prior range of [0, 0.08] in order to preclude negative values for low values of *k _CA_*.

The values for all parameters are either established from the literature ( Table 1) or fit (see below).

### National transmission (between-OA movements)

Between OAs individuals move daily across a network of locations defined by Scottish Census data adjusted by Google mobility data.

From the current population estimates we draw the number of individuals whose primary residence is mapped onto an OA, with their age group. The total population of Scotland from this estimate is 5,438,054 (Young: 919,580; Adult: 3,492,421; Elderly: 1,026,053). Of the adults 1,960,712 commute to work (reduced to 647,034 under lockdown (see details below).

An individual’s workplace is assigned by distributing a proportion of the population of each location to each work location, weighted by the proportion of individuals from each home location in the census flows data who work in another location. For each origin *o* and destination *d* we assign a weight *w _od_* from the census flow data:


$$w_{od}=\frac{n_{od}}{t_{o}}$$

where *n _od_* is the total number of people who move from *o* to *d* to work, and *t _o_* is the total number who move from origin *o* to any location for work. We take the individuals of each home location if they are eligible to work (total *n _o_*); in this case we assume all individuals of adult age 16–65. Each destination is assigned to *n _o_* × *w _od_* of these individuals. The individuals who remain have no assigned workplace—either they do not work, or they work within their home location.

For each day of the simulation we consider two time steps: a day step where individuals can move to their place of work, and a night step where those individuals move back to their home location. In each day step, we take each destination location *d*. Let *λ _d_* be the number of eligible workers who may move to the destination location. For each day the sampled number who move *s* is drawn from a Poisson distribution: *s* ~ *Poisson*( *λ _d_*). The sampled number of moves *s* is then scaled according to the percent change in mobility *m* (see below) for the given day: *s _m_* = ⌊ *s*(1 + $\frac{m}{100}$ )⌋.

In order to improve the computational efficiency of the simulation, movements of commuters between OAs were batched into groups of five, with movements between OAs of fewer than five individuals per day retained at a proportionate rate by drawing from a binomial distribution: *s _mt_* ~ *B*( *s _m_*, $\frac{1}{5}$ ). If the sampled number of workers *s _mt_* is less than or equal to the number of workers who may normally move to destination *d*, then those who move are sampled randomly from those who may normally move. However, if *s _mt_* is greater than the number of workers who may normally move to *d*, then the additional workers are drawn randomly from workers who have no assigned destination location. While this reduces the overall network link density, the effect on transmission dynamics is expected to be small. We note that this means that interpretation of the combined *β _D_* and *β _N_* must be made with caution.

For each night of the simulation, the workers who moved in the day step are moved back to their origin location.

### Modelling lockdown

**Figure 3.**
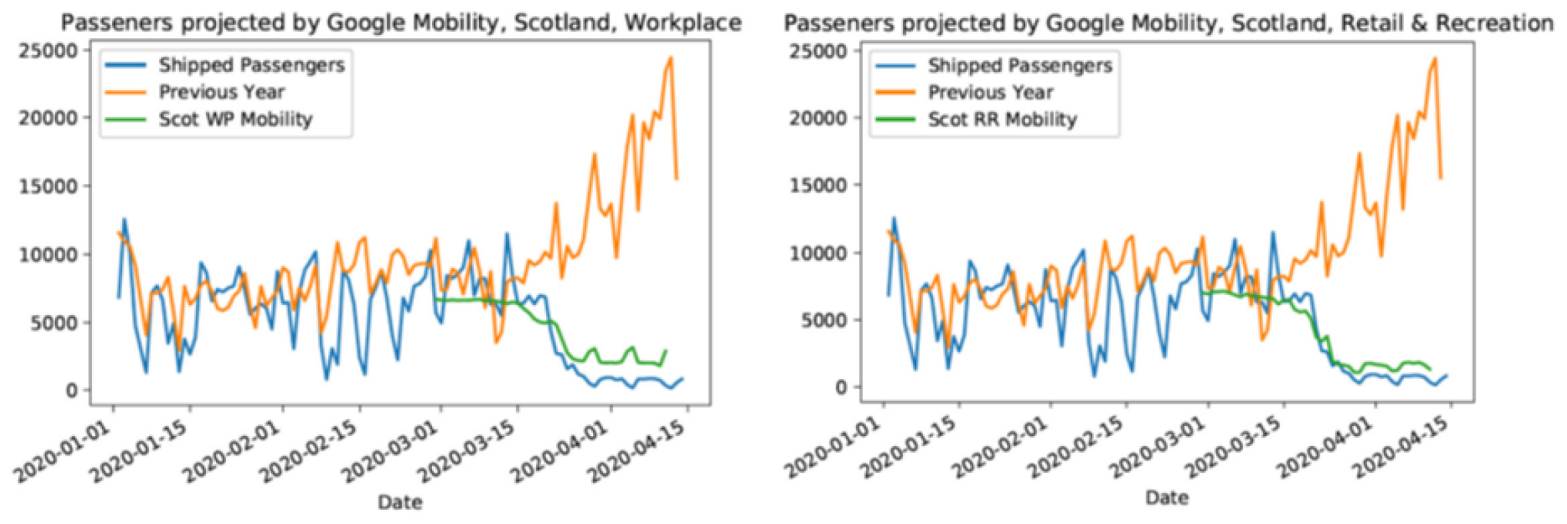
Comparison of Google mobility data for Scotland to CalMac Ferry records. Comparison to workplace mobility (left) Comparison to Recreation mobility (right). The comparison is relative to the mean value prior to lockdown on March 23rd, 2020. In order to provide confidence that inclusion of mobility reductions across regions is appropriate, we assume that urban areas such as Glasgow and Edinburgh are likely to be well represented, but that rural areas may be less so. To check this, we compare an independent dataset on independent sailings and passenger numbers for ferry services run by Caledonian MacBrayne, who operate all ferry services in the west of Scotland. A comparison of data from 2019 to 2020 and to Google Mobility data, shows a strong fidelity between the two datasets, as well as a substantial reduction in activity at point of lockdown. The similarity prior to lockdown between 2019 and 2020 also suggests that patterns of increased summer activity are unlikely to have had strong influences on our assumptions regarding commuter movements, at least in this area.

To model reductions in activity that are the aim of lockdown, we consider two factors. First, we thin movements in the simulation (mobility reduction) in proportion to the observed changes in mobility ^7^ (as above), also checking consistency with social contact surveys ^25^. This was corroborated for remote areas, where mobility data are few, with an independent dataset (see Figure 3). Second, physical distancing is incorporated via a reduction in contacts applied to both daytime and nighttime transmission rates (transmission reduction). For the post-lockdown period, we contingently fit a reduction in transmission only, assuming that posterior distributions for all other parameters estimated based on the pre-lockdown fit remain relevant, and considering a range of values consistent with independent estimates of the reproduction number *R _t_* ^8^.

### Model inference and computation

Simulated epidemics are compared to the spatio-temporal pattern of COVID-19 spread in Scotland. Non-observable parameters were estimated using the number of deaths where COVID-19 is recorded, considering all weeks beginning 9th March and ending on the 12th April 2020. Recorded deaths are from the weekly PHS records identified by the DZ of residence ^9^. These are the most complete and unbiased indicators of infection available during the early epidemic. However, they differ from other official sources as they record the date of registration of death. We assume that for each reported case, death occurs in the week prior to registration, which is allowed up to 8 days postmortem.

Estimation was performed using a Sequential Monte Carlo implementation of Approximate Bayesian Computation (ABC-SMC) ^26, 27^. We calibrated the model output to the cumulative weekly number of deaths due to COVID-19 aggregated at the level of CAs, using this spatial variation in deaths across Scotland to provide the necessary signature to properly calibrate the role of human mobility. Preliminary attempts to fit the data using weekly incidence had poor results (results not shown). We hypothesise that this is a result of a combination of very few deaths per CA to constrain the fits in the early stages of the epidemic, and also the complications from the multiple changes in the control efforts especially through March. Thus we chose to emphasise the later stages using as our observation the cumulative number of all COVID-19 related deaths per CA.

Simulated and observed summary statistics are compared via a score equal to a sum of squared errors, recorded weekly:


$$\begin{array}{l} score=\sum_{week}\sum_{CA}(\left(D_{sim}-D_{obs})(max(D_{obs}^{Z})\right){)}^{2}\\ \;+\sum_{week}\sum_{CA}(\left(D_{sim}^{Z}-D_{obs}^{Z})(max(D_{obs})\right){)}^{2} \end{array}$$

where *D _sim_* is the cumulative number of deaths per CA simulated and *D _obs_* its observed value, $D_{sim}^{Z}$ is the cumulative number of data zones with deaths per CA simulated and $D_{obs}^{Z}$ its observed value. *max*( *x*) is the maximum number of *x* over all CAs, used to balance the relative contributions of the two elements of the metrics to the inference. The total number of infected individuals at the start of the simulation (the seeds) are fitted as part of the inference. The seeds are randomly assigned a disease state from *E*, *I ^M^*, and *I ^S^*. Seed locations are stochastically assigned according to the cumulative proportion of deaths registered per Intermediate Zone, as recorded up to the week of March 23rd, 2020. Intermediate zones are aggregates of approximately five DZs; this scale is chosen to account for clustering of infections in areas near to identified deaths.

Uniform prior distributions constrain all parameter values to plausible ranges based on the available literature relevant to the early, pre-lockdown period. Infection dynamics are simulated via a *τ*-leap algorithm using halfday timesteps ^28^. All parameters are listed in Table 1. The inference framework is run on a distributed application framework (Akka) ^10^ running on a cloud computing infrastructure (Amazon AWS2) ^11^. The model code has been written using industry grade software engineering practices including agile development for project task planning, test driven development, pair programming and code reviews to produce unit tested, robust, and reusable software components. The majority of the code has been reviewed by a second software developer.

## Results

### Health index and mortality

The results of univariable analyses of the SIMD variables for the population level is presented in Table 2. As access has the lowest Akaike information criterion (AIC) and Bayesian information criterion (BIC) and is orthogonal to the other variables we tested it against the remaining variables in turn to check for a multivariable model. The model with access and health had the lowest AIC (276.3), BIC (282.2) and low variance inflation factor (1.291) and was not overdispersed ( Table 3). We analysed death records from Public Health Scotland (PHS) data on non-care home deaths due to COVID-19. We examined the impact of different deprivation factors relevant to this period; whilst deprivation overall is significantly associated with increased COVID-19 mortality, this could be further disaggregated:

1. Population level risk of COVID-19 mortality is associated with the SIMD indicator that describes (good) accessibility and orthogonally with the SIMD indicator that describes (poor) health. Indicating that areas with poorest health and good access experienced higher COVID-19 mortality.
2. Risk of excess COVID-19 mortality (COVID-19 deaths as a fraction of all deaths) is most closely associated with the access indicator component of SIMD. This indicates that the areas that have good local connectivity and transport will have higher rates of COVID-19 transmission.
3. We hypothesise that access is a proxy for earlier introduction of infection and the mobility patterns in our data. Thus the observed differences in mortality due to access is already accounted for in our model (via the initial seeding plus transmission dynamics). We therefore fit a modifier to COVID-19 mortality considering the average health index (only) in each CA.

**Table 2.** Results of the univariable model with Covid deaths / population as the outcome. SIMD, Scottish Index of Multiple Deprivation; AIC, Akaike information criterion; BIC, Bayesian information criterion.

Predictor	Estimate	St. error	p-value	AIC	BIC
Population / 100000	0.0137	0.067	1.063	303.2	307.6
SIMD score	0.046	0.012	<0.001	291.5	295.9
SIMD education indicator	0.036	0.013	0.005	297	301.4
SIMD health indicator	0.042	0.008	<0.001	283.1	287.5
SIMD housing indicator	0.025	0.009	0.006	297.4	301.8
SIMD access indicator	-0.04	0.009	<0.001	283.8	288.2
SIMD crime indicator	0.049	0.013	<0.001	290.7	295.1
SIMD income indicator	0.04	0.009	<0.001	289.8	294.2
SIMD employment indicator	0.041	0.009	<0.001	287.2	291.6
SIMD score (exc access)	0.047	0.011	<0.001	288.2	292.6
$\sqrt{\text{population density}}$	0.017	0.006	0.003	295.9	300.3

**Table 3.** Results of the multivariable model with Covid deaths / population as the outcome. SIMD, Scottish Index of Multiple Deprivation.

Variable	Estimate	St. error	p-value
Intercept	-7.91	0.320	<0.001
SIMD access indicator	-0.024	0.008	0.004
SIMD health indicator	0.029	0.008	<0.001

### Movement and network patterns

**Figure 4.**
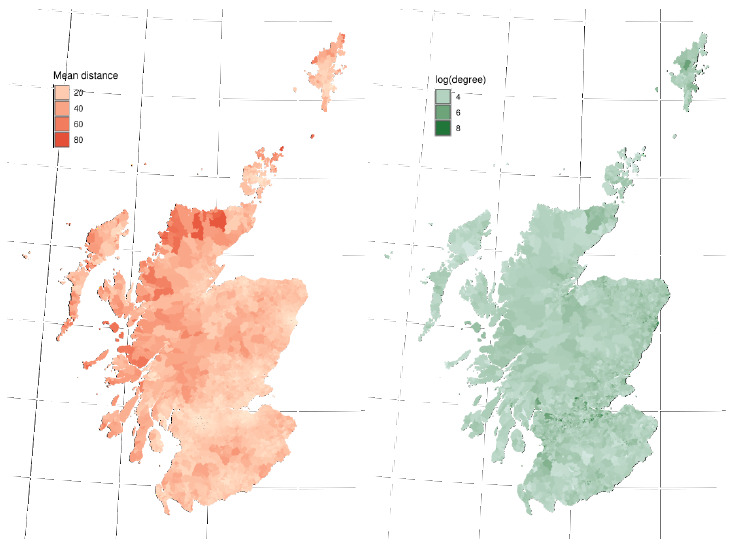
Commuter movement patterns, with commuters aggregated by Output Area (OA) (Census areas with typically 50–500 individuals, maximum of 2081). Data according to the 2011 census. Mean distance travelled from OA in km (left) and mean number of OA’s to which each OA is connected to (right). The greatest distances are travelled on average by individuals in remote locations. The greatest network degree is found in highly urbanised areas.

The pattern of movement generated at the OA level shows substantial differences in both the average distance travelled, and the connectedness between OAs across the country. Individuals in remote areas move the farthest to work on average, and individuals in the densely populated “Central Belt” are the most connected ( Figure 4).

### Parameter posteriors and model fit

**Figure 5.**
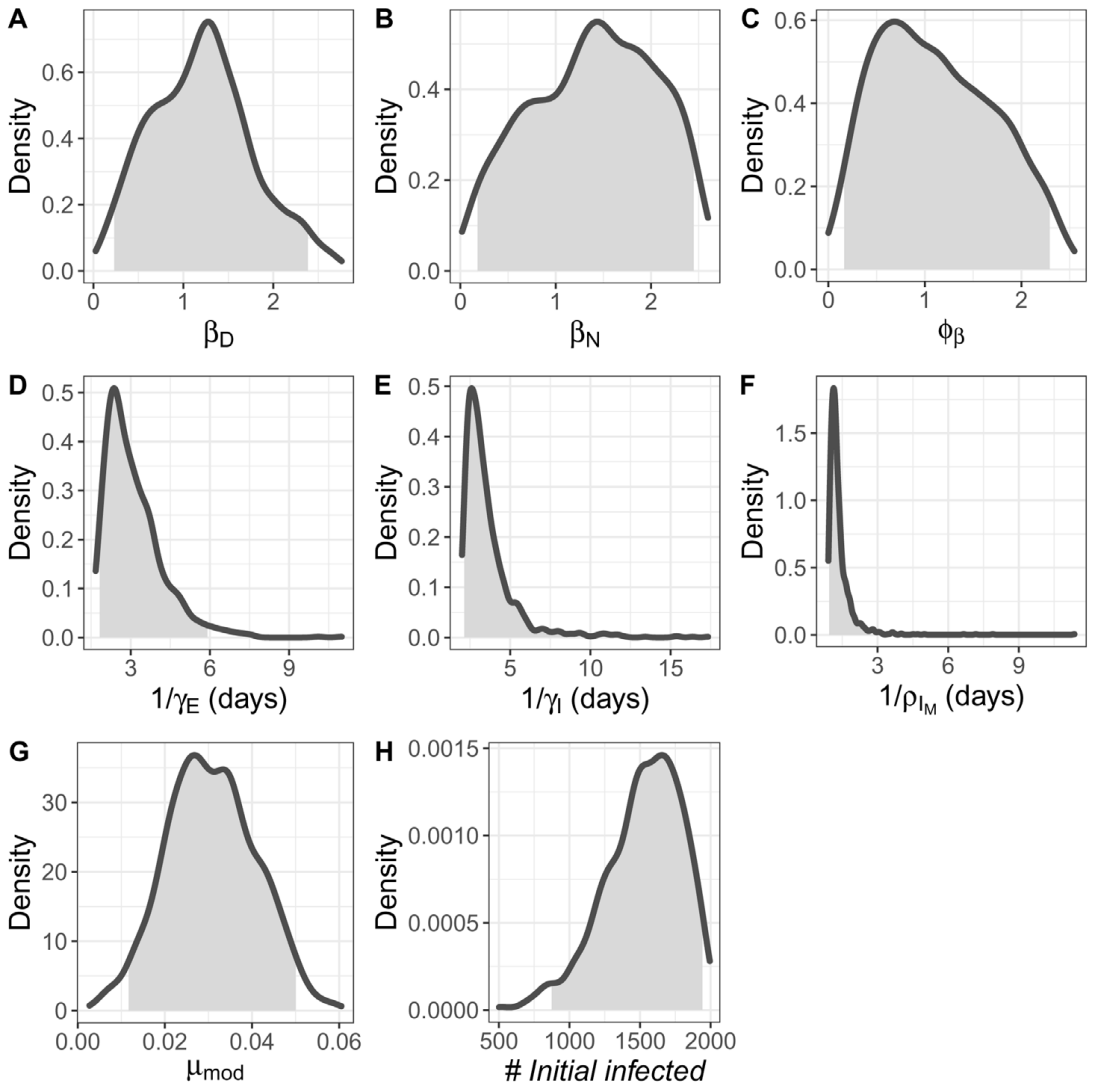
Posterior distributions of fitted parameters. From top left to lower right: ( **A**) frequency dependent transmission rate for severely infectious individuals in daytime locations (per five severely infectious individuals, per half day), ( **B**) frequency dependent transmission rate for severely infectious individuals in nighttime locations (per severely infectious, per half day), ( **C**) multiplier for mildly infectious individuals. ( **D**) duration of the exposed stage of infection (in days), ( **E**) duration of the mildly infectious period in the absence of recovery (in days), modifier ( **F**) time to recovery for mildly infectious individuals in the absence of progression to severely infected (days) ( **G**) mortality rate modifier, and ( **H**) number of seed infections. Shaded areas show 95% credible intervals for all parameters.

**Figure 6.**
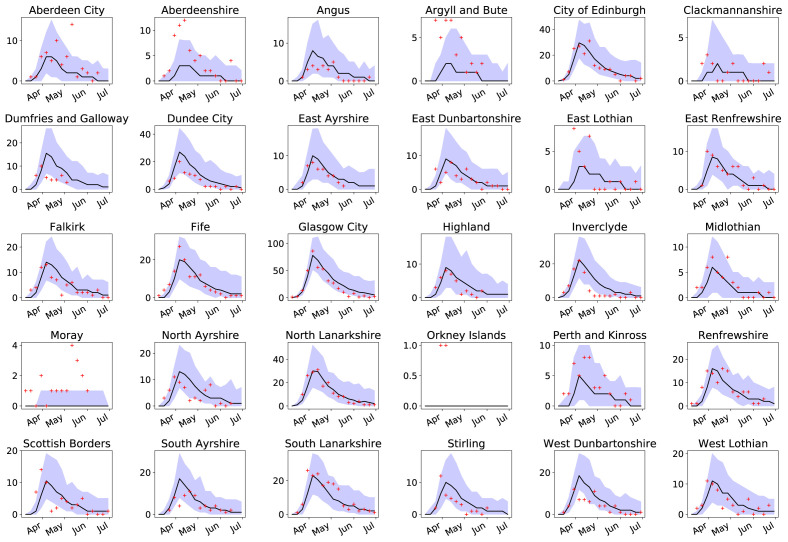
Number of deaths per week for all Council Areas in Scotland (bar Shetland). Incidence number (red crosses) of coronavirus disease 2019 related deaths compared to the median of 100 simulations (black line), and 95% confidence intervals (purple areas).

**Figure 7.**
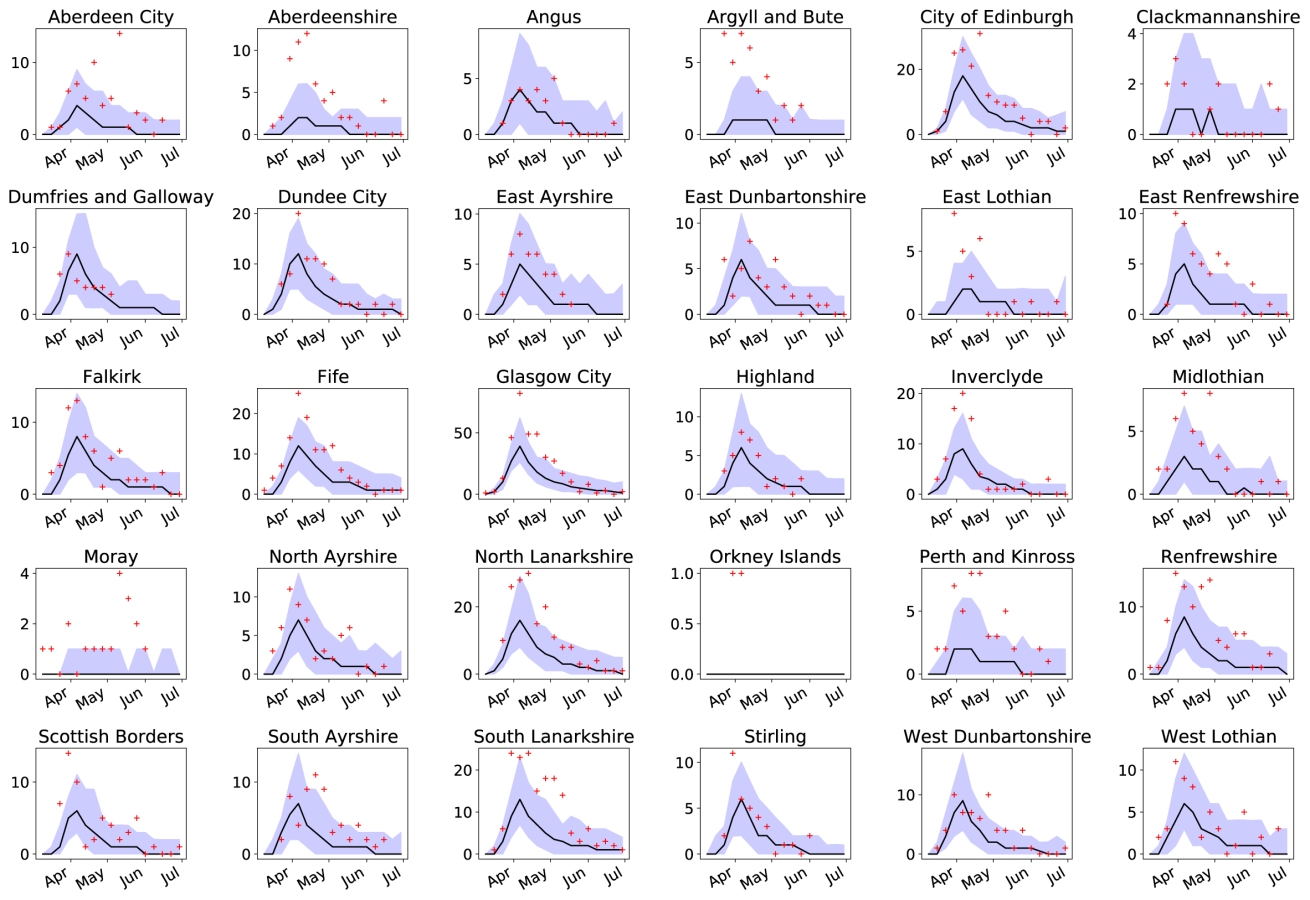
Number of data zones (DZ) with deaths per week for all Council Areas in Scotland (bar Shetland). Incidence number (red crosses) of DZs with coronavirus disease 2019 related deaths compared to the median of 100 simulations (black line), and 95% confidence intervals (purple areas).

In the fitted model, parameter posteriors are strongly unimodal ( Figure 5) with only weak pair-wise correlations. The most likely (mean) estimate for the mortality modifier is *μ _mod_* = 0.03, resulting in COVID-19 mortality rates that are 2.45× higher in the CA with the worst health index, compared to the best. Post-lockdown, the best fit value (lowest score) occurs when transmission rates are reduced to 0.12× the pre-lockdown value, starting from 28th March. The number of observed deaths per CA per week mostly lie well within the range of 95% of the fitted simulations (parameters drawn in sets from the posterior parameter distributions; see Figure 6). The number of DZs with deaths is more likely to exceed these limits ( Figure 7) though with low numbers and therefore greater stochastic variability in the data.

### Impact of distance reduction and transmission reduction on COVID-19 spread

**Figure 8.**
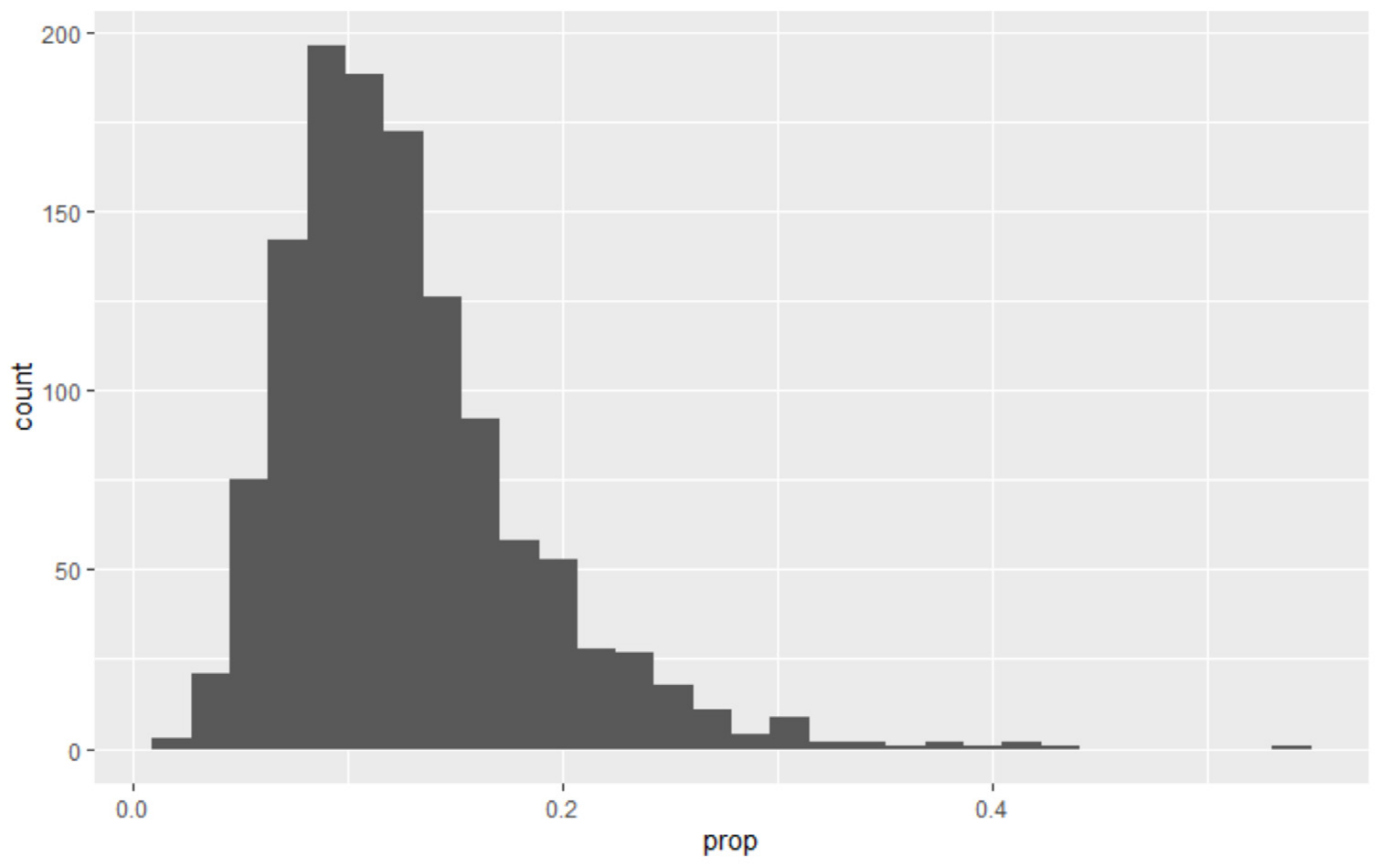
Proportion of transmission occurring outside of Output Area (OA) of residence, across all OAs in Scotland. For most OAs the majority of transmission is estimated to occur within the home location.

Lockdown restrictions impact both the local spread of infection via physical distancing measures that change transmission rates and geographical spread (e.g. reductions in travel-to-work). In only a few cases over 25% of infection occurs outside the OA of residence ( Figure 8).

**Figure 9.**
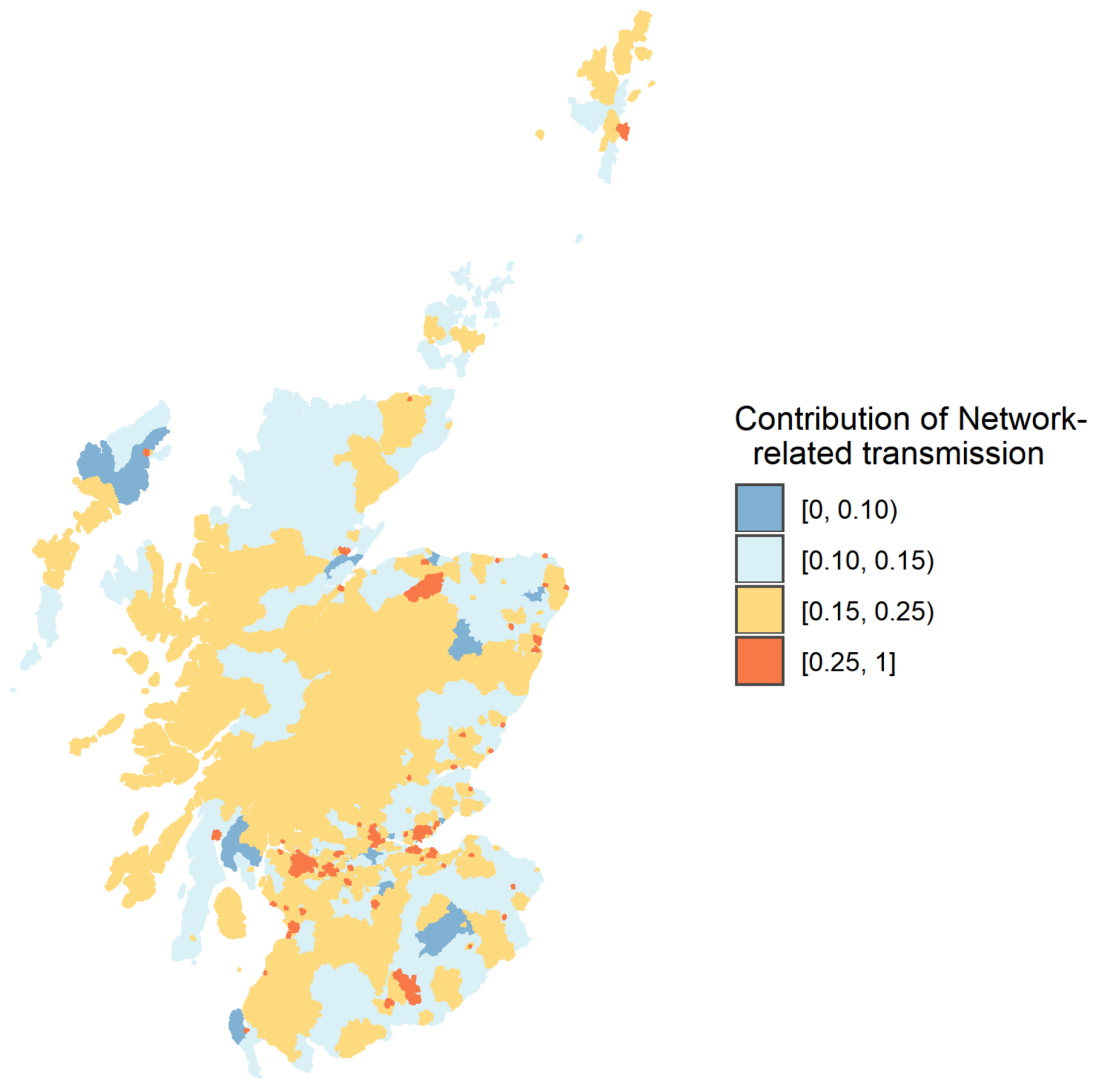
Estimated relative proportion of transmission events likely to occur within the Output Areas of residence of a single infected individual introduced into an otherwise susceptible population. Areas in yellow (high) and orange (extremely high) generally represent highly urban areas, with considerable inward commuting traffic.

These are typically urban areas, most likely with considerable inward commuting traffic ( Figure 9). However, disease spread is predominantly local, with the most likely outcome being more than 90% of infection occurring within OA.

**Figure 10.**
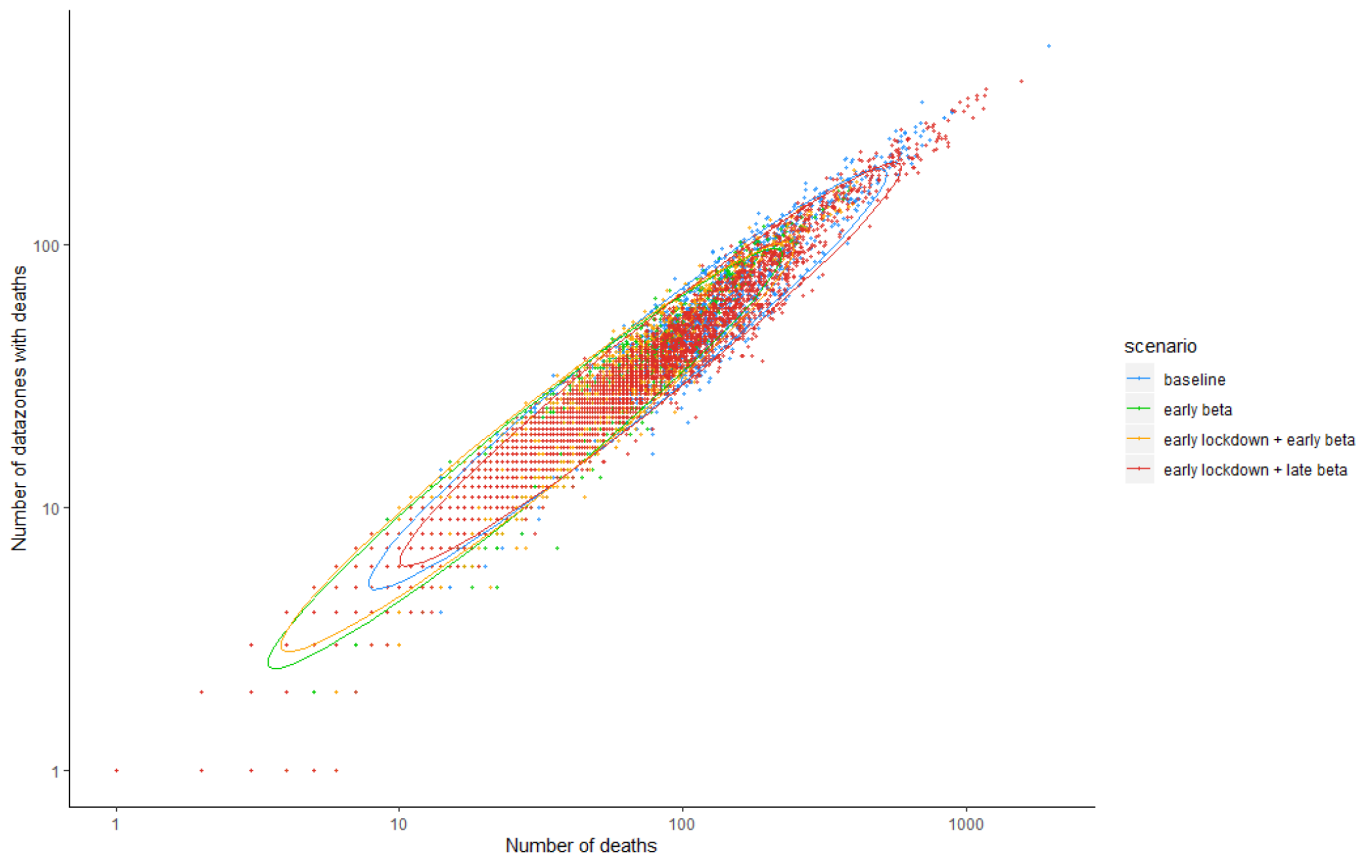
Comparison of number of coronavirus disease 2019 related deaths and number of data zones with deaths as of April 26th 2020, contrasting baseline (imposition of restrictions as they occurred), early (March 9th) imposition of physical distancing measures but without restriction of long distance travel (early beta), early imposition of long distance travel restriction only (early lockdown) and early imposition of both.

**Figure 11.**
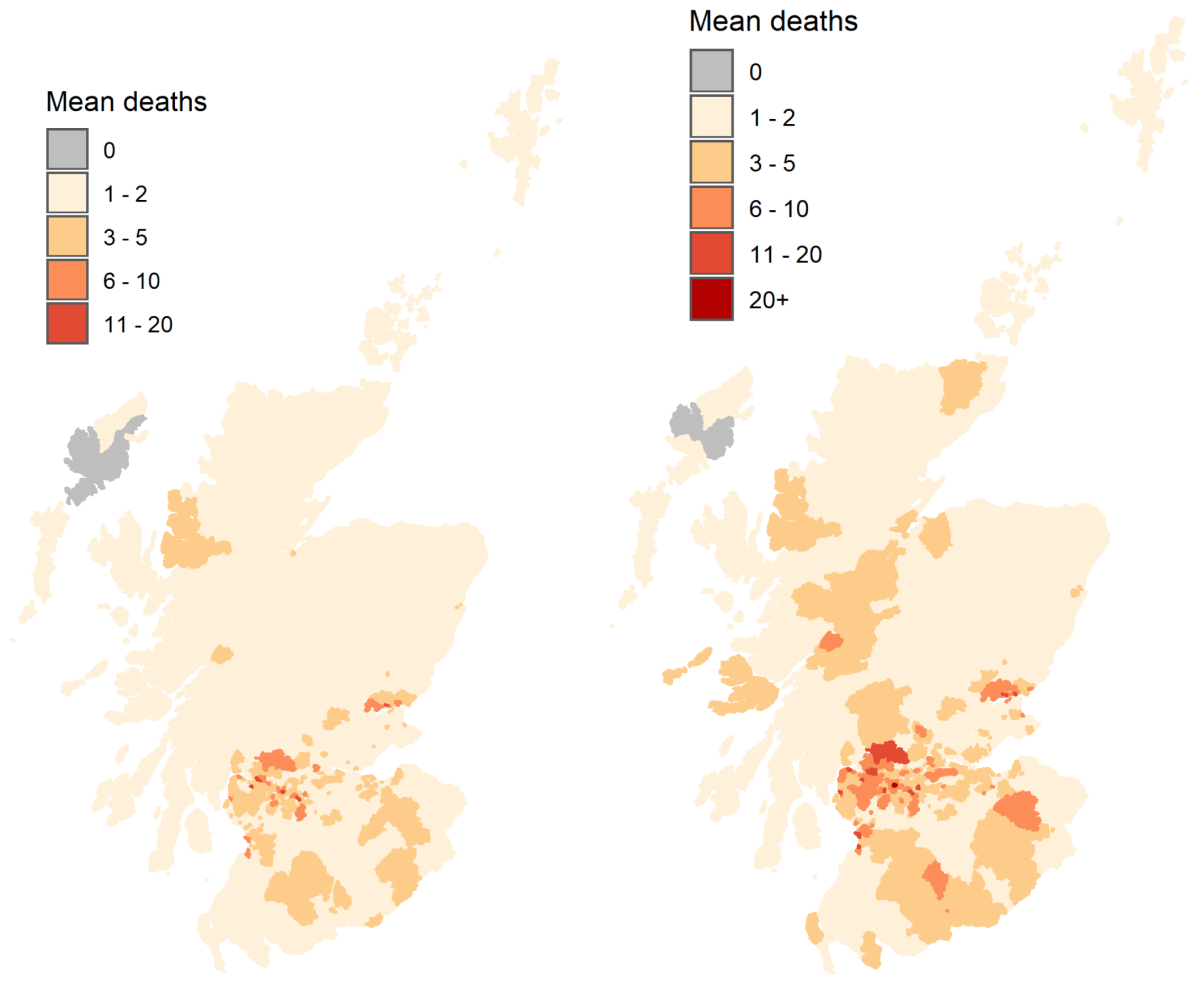
Mean number of deaths per Output Area, averaged over 50 simulations for early lockdown on 16th March 2020 (left) and lockdown as it occurred on 23rd March 2020 (right) considering the total number to 30th April, 2020.

We consider counterfactuals where some lockdown restrictions are imposed on 9th March, two weeks prior to the actual date, and just before the first death due to COVID-19 in Scotland was reported. The reduction in transmission rates is attributable to physical distancing, or reduction in mobility, or both. With earlier lockdown, we predict a median 581 deaths (95% of simulations within 377 to 1,010 deaths) by 26th April 2020, compared to 2,722 (95% of simulations within 1,294 to 4,050) in the baseline scenario (observed number is 2,795, assuming that all deaths occur in the week prior to the week the death is registered in). Most of this difference is due to the reduction in transmission rates ( Figure 10). The reduction in the total number of deaths also results in a reduction in geographical spread with many fewer DZs affected by COVID-19 mortality in the early lockdown scenario ( Figure 11).

## Discussion

Exploiting our estimates of the relative contribution of longer distance movements to work and local transmission that include work and non-work interactions, we show that at this early stage physical distancing had a dominant impact on the death rate, with little evidence that longer range movement restrictions were important for reducing deaths.

Our model is fitted to the spatio-temporal pattern of deaths due to early stage COVID-19 under the assumption that it maps well onto the patterns of infection at this spatial scale and at this stage of the epidemic. While an inference done on case distributions would be a more direct approach to estimating transmission parameters, testing and hence case ascertainment in the early stages of the pandemic was poor. Our modelling approach shows that, even though the spatio-temporal pattern of deaths is subject to substantial stochastic variability, it can be used to generate meaningful opinions in our posterior parameter estimates and form the basis for prediction of counterfactuals.

Movement restrictions are of course critical for preventing the seeding of new areas and therefore a direct estimate of its role in preventing spread at these early stages of the epidemic can provide vital insights into how important rapidly applied measures are and what elements of those measures are most important. Here we show that a movement ban on its own, while it would restrict the important outcome of geographical spread would on its own have had minimal impact at this stage, on the scale of the epidemic. Instead, local physical distancing measures would have had the greatest impact.

Our analysis does not preclude increased transmission due to deprivation at the local level (e.g. due to greater use of public transport, for example) as these factors are not embedded in the model. Of course over the longer term, both elements of long distance travel restrictions and transmission reduction are needed; reductions in both the areas being affected (allowing for potentially more targeted control efforts) and total deaths (both directly saving lives, and reducing impact on the health care system). More localised or clustered infections also will ultimately result in a higher proportion of recovered individuals in hotspot areas, promoting localised herd immunity effects. Further, as these hotspots were typically in more urban areas, this also has the important consideration of reducing infection rates in remote areas, where access to hospitals and intensive care units (ICUs) is typically poor. Of course, not all long distance movements occur for work purposes and should the patterns of non-work movements be substantially different to movements-to-work patterns, and be of sufficient volume, then it could have an influence on our results. However, the available mobility data suggest that national lockdown restrictions would have had a greater effect on at least the volume of non-work movements.

The best parameter estimates give a high probability that mildly infected individuals have higher transmission rates than the severely infected, though with shorter duration. This does not imply that they are necessarily producing more virus, as those who have severe symptoms may be self-isolating or be more strictly physically distancing. Our model also estimates the number of individuals with infection in early March, with the most likely case being on the order of 1500 infected individuals in Scotland at this time. While our approach is crude and does not take account of continuing importation of individuals over time, it is substantially greater than the estimate of 113 introductions of COVID-19 into Scotland based on viral sequence data ^29^, but is plausible if one takes into account the additional infections these introductions would have caused by the start of our simulations.

By explicitly modelling transmission dynamics, this allows us with relatively few data, to infer in our model that health-related deprivation results in a 2.45× difference in the death rate due to COVID-19 across CAs, rather than because these areas carried a greater burden of infections earlier in the pandemic. Deprivation (including health) in Scotland varies substantially at a much finer scale, with zones with the highest deprivation often neighbouring zones with the lowest. This suggests that, were the data available, a deeper interrogation could provide a much more refined assessment of potential health burdens and risks associated with geographical spread.

The effectiveness of lockdown will vary in space and time, due to differences in human behaviour, and also because of non-linear relationships between the numbers of cases, probability of spread, and logistical burdens on care homes, hospitals and ICUs reduced. These potentially counterbalancing factors would of course have to be considered in more detail for a full understanding of lockdown effectiveness. Despite these caveats, our simple approach is useful to strategically examine trade-offs between travel related restrictions, and physical distancing when evaluating future releases from lockdown. Here however, most of the protection from new infections is a result of transmission reduction; long distance restrictions only have a minimal impact because in our model, as overall infection pressure is conserved—i.e. if it does not occur in work locations, it is assumed to occur at home. Thus only extreme travel restrictions are likely to have an impact, at least until build-up of immunity levels is more substantial than observed at this early stage.

Since this analysis, the successful deployment of COVID vaccines in the UK and elsewhere has meant that the response to the current pandemic need not include such extreme measures. However, where vaccine escape mutants become a problem of sufficient concern, and booster vaccines not be readily available, the need for future extreme controls cannot be ruled out, with the recent spread of the Omicron variant one example, although the most extreme measures have thus far proven unnecessary. In situations where such measures are considered, our results indicate that even extreme lockdown measures do not entirely prevent geographical spread between regions; thus any restrictions must include measures to not just reduce mobility but also reduce transmission if, in the future, we are to prevent the spread of COVID-19 to areas that have successfully eradicated local COVID-19 cases.

## Data availability

### Underlying data

Data on underlying population and movements: NRS Census Flows data, Table WU01UK Available from: https://wicid.ukdataservice.ac.uk Access to Output Area level data requires an academic registration.

Data on human mobility over the simulation period: Google Mobility Reports Available from: https://www.google.com/covid19/mobility/

Data on deprivation-related demographics of underlying population: Scottish Index of Multiple Deprivation (SIMD) 2020 Available from: https://www.gov.scot/collections/scottish-index-of-multiple-deprivation-2020

Data on COVID-19 testing and mortality (at Datazone level) were provided by Public Health Scotland (PHS) and made available via the PHS Electronic Data Research and Innovation Service (eDRIS). Available from: https://www.isdscotland.org/Products-and-services/Edris/ The sensitive nature of these data required a Data Sharing Agreement between PHS and University of Edinburgh, and therefore are not publicly available.

### Extended data

Model code available from: https://github.com/Kao-Group/SCoVMod.git

Archived model code at time of publication: https://doi.org/10.5281/zenodo.6420991 ^30^

License: MIT

## Footnotes

^1^ https://statistics.gov.scot/

^2^ https://www.isdscotland.org/Products-and-Services/EDRIS/COVID-19/

^3^ https://wicid.ukdataservice.ac.uk

^4^ https://www.scotlandscensus.gov.uk/about/2011-census/2011-census-geographies/, https://www.nrscotland.gov.uk/files/geography/2011-census/geography-bckground-info-comparison-of-thresholds.pdf

^5^ https://www.gov.scot/collections/scottish-index-of-multipledeprivation-2020/

^6^ https://www.google.com/covid19/mobility/

^7^Google Community Mobility Reports: https://www.google.com/covid19/mobility/

^8^COVID-19: Framework for Decision Making, Further Information (published 23rd April, 2020), accessed 11th May, 2020: https://www.gov.scot/publications/coronavirus-COVID-19-framework-decision-making-further-information/

^9^Data provided by eDRIS is subject to data sharing agreement due to potential for individual disclosure.

^10^ https://akka.io

^11^ https://aws.amazon.com
