# Supplementary material for: SCoVMod – a spatially explicit mobility and deprivation adjusted model of first wave COVID-19 transmission dynamics
Source: Wellcome Open Res. 2022 May 26;7:161. [Version 1] doi: 10.12688/wellcomeopenres.17716.1 (PMC9274017; doi:10.12688/wellcomeopenres.17716.1)
Supplement: Peer review history

# Peer review: Reviewer response for version 1

**DOI:** 10.21956/wellcomeopenres.19604.r50789

**Published:** 2022-07-11

**Authors:** Cameron Zachreson

**Affiliations:** 1 School of Computing and Information Systems, The University of Melbourne, Melbourne, Vic, Australia

This paper presents a spatially explicit model of COVID-19 in Scotland. The population model incorporates medium to long-range mobility patterns as well as local contact rates and the study investigates the effectiveness of lockdown measures with respect to these two model components. The study makes the case that, in Scotland, mobility restrictions were relatively ineffective compared to the influence of local transmission mitigation (i.e., reduction in contact rates within regions). The results indicate that this is because mobility restrictions were implemented too late, and were not stringent enough to prevent the spread of SARS-CoV-2 between areas. The conceptual case is clear and the argument is reasonable, but there are some strong assumptions that could be tested to make the conclusions more robust. There are also some minor clarity issues with the model description, and the presentation of data could be more thorough/professional. Overall I found the paper well-written, the arguments clearly articulated, and the conclusions relatively convincing. Furthermore, the code provided appears to have been developed according to a high professional standard (though it could benefit from additional explanatory documentation and inline comments). 

**Main issues:** strong assumption about initial conditions; posterior correlations not presented. 

 Suggest analysis of sensitivity to strong assumptions: 

- (i) distribution of initial cases in proportion to observed cumulative deaths (this one is the most important):
  Models like this are very sensitive to initial conditions (location and number of seed cases). Since fitting is performed based on the spatial distribution of deaths, the assumption that the initial conditions are themselves strongly dependent on the observed outcomes could be an artificially strong constraint. The true initial conditions were not observed with high levels of accuracy, so some well-reasoned relaxation of the strong constraint used in the design of initial conditions should be explored. For example, a null model could be compared in which the seed cases are distributed with respect to population only (which could be age-stratified etc.). 
- (ii) Distribution of mobility reduction uniformly in proportion to global mobility reduction:
  The model defines mobility patterns based on a statistical snapshot. The mobility dynamics under lockdown are treated as uniform adjustments to the snapshot of mobility patterns in the base model. This is a reasonable treatment given the limitations of the data, but represents a strong assumption against which the robustness of the results should be tested. For example, a perturbation approach could be used to systematically randomise the pairwise reductions in mobility, or an alternate assumption could be tested, in which relative mobility reductions depend on distance between origin and destination or some other set of region pair properties. 

I suggest additional results:

- (i) Correlations in joint posterior (for parameters in Figure 5) would help informed readers understand model uncertainty more clearly. 
- (ii) Full posterior and correlations for estimated efficacy of physical distancing with epi parameters would help quantify uncertainty in the conclusions. Currently, only the best-fit value is reported (0.12). 

**Minor issues: **

- I found the model software well organised, but it could benefit from more informative documentation and commenting, as the design is relatively complex. 
- Figures need a thorough proof-read and should be presented in high-resolution format (preferably vector graphics)
- The results in Figure 10 could be presented more clearly (a 1-d histogram for each observable may be preferable given the very high correlation). 
- Equations should be numbered.
- Model could be more clearly described as an agent-based model, and specified according to agent state variables (Note: this is especially confusing in the discussion of how individuals are assigned work locations and how the mobility flows are computed - it needs to be clear that each working adult is assigned only one location, I had to read the section four times to understand that origins were not stochastically assigned each day).  

Is the work clearly and accurately presented and does it cite the current literature?

Yes

If applicable, is the statistical analysis and its interpretation appropriate?

Yes

Are all the source data underlying the results available to ensure full reproducibility?

Yes

Is the study design appropriate and is the work technically sound?

Yes

Are the conclusions drawn adequately supported by the results?

Yes

Are sufficient details of methods and analysis provided to allow replication by others?

Yes

Reviewer Expertise:

Computational modelling of disease transmission, with a focus on agent-based models of pandemics in structured populations.

I confirm that I have read this submission and believe that I have an appropriate level of expertise to confirm that it is of an acceptable scientific standard.

# Peer review: Reviewer response for version 1

**DOI:** 10.21956/wellcomeopenres.19604.r51159

**Published:** 2022-07-07

**Authors:** Christopher Davis

**Affiliations:** 1 Mathematics Institute, University of Warwick, Coventry, UK

The authors present a spatial agent-based transmission model for COVID-19 in Scotland, which is applied to the early months of the pandemic in 2020. The model parameters are obtained by a combination of using values from previous literature, values taken directly from data, and estimation using ABC-SMC. Mortality in the model is adjusted spatially using data from the Scottish Index of Multiple Deprivation.

 The model is used to estimate the impact of lockdown restrictions, the difference in mortality by deprivation status, and, given the model is spatially structured, assess the separate impact of long-distance movement restrictions and local transmission reduction. 

 Overall, the manuscript is well written and explicit in the assumptions made to arrive at the presented conclusions. The message is clear and the methods are appropriate to tackle the questions asked. The model is shown to have a good fit to the death data. All data used is listed and the model code is available to access.

 Some minor comments are listed below:

- In the first paragraph “lockdown” and “key workers” are italicised, without being defined anywhere else.
- Table 1 shows the fitted parameters, but the parameter symbols do not completely match those used for the posteriors in Figure 5 (e.g. the transmission rate multiplier for mild infectors is given by both “y” and “phi_beta” and differences in capitalisation for day and night).
- For the equation for the number of deaths, should it also include the number of susceptible people?
- The definition of the degree in Figure 4 is not completely clear.
- In Figure 10, the y axis label should have “data zones” as two words. The caption should be split into two sentences, and the number of simulations done also stated here. The methodology for drawing the rings is not obviously explained. In addition, most of the points look red (all the red points were plotted last?), and so the figure is difficult to read. Could some jittering be applied or the order in which the points are plotted randomised to provide a more representative picture?
- In Figure 11, the first of the two figure legends should be removed.
- Also, Figure 3 and Figure 4 are currently presented as low resolution images.

Is the work clearly and accurately presented and does it cite the current literature?

Yes

If applicable, is the statistical analysis and its interpretation appropriate?

Yes

Are all the source data underlying the results available to ensure full reproducibility?

Yes

Is the study design appropriate and is the work technically sound?

Yes

Are the conclusions drawn adequately supported by the results?

Yes

Are sufficient details of methods and analysis provided to allow replication by others?

Yes

Reviewer Expertise:

Mathematical epidemiology

I confirm that I have read this submission and believe that I have an appropriate level of expertise to confirm that it is of an acceptable scientific standard.
